# Obstetric, maternal, and neonatal outcomes in COVID-19 compared to healthy pregnant women in Iran: a retrospective, case-control study

**DOI:** 10.1186/s43043-021-00059-2

**Published:** 2021-06-14

**Authors:** Seyed-Abdolvahab Taghavi, Solmaz Heidari, Shayesteh Jahanfar, Shakiba Amirjani, Amireh Aji-ramkani, Maryam Azizi-Kutenaee, Fatemeh Bazarganipour

**Affiliations:** 1 Social Determinants of Health Research Center, Yasuj University of Medical Sciences, Yasuj, Iran; 2 Fertility and Infertility Research Center, Hormozgan University of Medical Sciences, Bandar Abbas, Iran; 3 MPH Program, Department of Public Health and Community Medicine, Tufts University School of Medicine, Boston, MI USA; 4 Department of Gynecology and Obstetrics, Yasuj University of Medical Sciences, Yasuj, Iran

**Keywords:** COVID-19, Pregnancy, Iran

## Abstract

**Background:**

The purpose of the present study was to evaluate obstetric, maternal, and neonatal outcomes in COVID-19 compared to healthy pregnant women in Iran.

**Results:**

A case-control study was conducted on 55 COVID-19 as the case and 55 matched control pregnant women in Hormozgan, Iran. Patients were considered to be cases if they had a positive COVID-19 test plus a positive chest X-ray result. Our measures were COVID-19 symptoms, including laboratory evaluations, clinical symptoms, and maternal and neonatal outcomes.

The most prevalent symptoms related to COVID-19 were fever (69.09%) and cough (58.18%). Less common symptoms included fatigue, diarrhea, shortness of breath, sore throat, and myalgia. Hydroxychloroquine/chloroquine (58.18%) and antibiotic therapy (45.45%) were the most prevalent management in COVID-19 patients. Based on our findings, maternal and obstetric outcomes—neonatal in case groups—such as mode of delivery, premature rupture of membrane, postpartum hemorrhage, perineal resection rate, neonates’ birth weight, Apgar score, and neonatal asphyxia rate were similar to pregnant women without COVID-19. We observed a higher incidence rate of premature delivery in COVID-19 cases (25 vs. 10%) (*p* < 0.05). In the present study, we found that women with COVID-19 had a more than twofold increased odds of preterm labor. History of preterm delivery was also associated with high twofold odds of preterm labor.

**Conclusion:**

We observed a higher incidence rate of premature delivery in COVID-19 cases. Women with COVID-19 had a more than twofold increased odds of preterm labor. Considering prematurity has high morbidity and is regarded as the primary cause of mortality in children under 5 years old, more case-control studies are needed to ascertain the results.

## Background

The current state of the COVID-19 epidemic around the world is intense and disturbing [1]. Coronaviruses are a family of viruses that can cause a wide range of illnesses, from the common cold to acute respiratory symptoms, and can cause death due to pneumonia and respiratory problems [2]. With the spread of the coronavirus, the incidence of pregnancy in mothers is also increasing [3]. Prevention and control of the disease in pregnant women and the potential risk of vertical transmission have become a significant concern [4].

Limited studies have been performed on their effect on pregnancy [5]. Since the monitoring systems for the COVID-19 have been created, it is essential that information about the severity of the disease and maternal and fetal outcomes collected and reported some preliminary evidence that can be used to guide the treatment of pregnant women with COVID-19 used to be presented. In pregnant women, due to weakened immune systems and the respiratory system (decreased diaphragm height, increased oxygen consumption, mucosal edema of the respiratory tract), their tolerance to hypoxia is reduced [1]. Respiratory problems are expected to increase in pregnant women with COVID-19. However, it should be noted that the potential risks of cytokine storm due to infection in pregnant women may be severe complications and even death [6]. Together, these factors make pregnant women, their fetuses, and their newborns more vulnerable to infectious diseases [1]. The consequences of coronavirus in pregnant women and their infants are unclear [7], and the results have been inconsistent [4]. Because most studies have focused on no pregnant people, studies on the effects of coronavirus on pregnancy and childbirth are very limited [8]. In a 2020 study by Zhang et al., which studied 16 pregnant women with COVID-19 and 45 healthy pregnant women, there was no significant difference in maternal weight, gestational age at birth, infant weight during labor, or maternal blood loss during surgery between groups [4]. But according to studies, mothers with COVID-19 are at risk for preterm birth [3, 4, 6, 8, 9], abortion [3, 9], premature rupture of membranes [1, 4, 8], preeclampsia [1, 9], cesarean [4, 7, 9], fetal distress [1, 4, 6], prenatal dead [9, 10], and admission in NICU [9, 10], and preterm delivery is known as the most common adverse outcome of pregnancy [9]. These complications can be attributed to viral infections as well as physiological changes that reduce a pregnant woman’s tolerance for hypoxia in late pregnancy [10]. Overall, a review of the results of studies shows that clinical signs, laboratory results, and radiographic criteria in pregnant women with COVID-19 are the same as in non-pregnant adults. Common manifestations of COVID-19 disease in pregnant women include fever, cough, and myalgia. The most common laboratory results are decreased lymphocytes, increased CRP in the blood, and complications of pregnancy and delivery include increased preterm delivery and cesarean section [3, 4, 6, 8, 9]. The number of studies conducted was very limited and generally case reports and most of them are from China. Therefore, there is a need for further studies in this field and in different countries of the world. The aim of this study was to compare obstetric, maternal, and neonatal outcomes in women with COVID-19 with healthy pregnant women.

## Methods

### Design and data collection

This was a retrospective case-control study. One hundred ten pregnancies were referred in Shahid Mohammadi Hospital, Bandar Abbas, Hormozgan, Iran, between March to November 2020. The Ethics Committee of the Hormozgan University of Medical Science, Bandar Abbas, Iran, approved the study. The study was a retrospective analysis of medical records and anonymized patient identities; thus, informed consent was not required.

According to Zhang et al. [4] (*α* = 0.05; *β* = 0.2; *P*_1_, fetal distress in COVID-19 group = 16%; *P*_2_, fetal distress in control group = 1.6%), sample size was estimated at 55 patients per group (a total of 110 persons).

$$ n=\frac{{\left({Z}_{1-\alpha /2}+{Z}_{1-\beta}\right)}^2\left[{P}_1\left(1-{P}_1\right)+{P}_2\left(1-{P}_2\right)\right]}{{\left({P}_1-{P}_2\right)}^2} $$

All of the pregnant patients with COVID-19 who were admitted to Shahid Mohammadi Hospital Hormozgan, Iran, were enrolled from March to November 2020.

Patients were considered cases if they had a positive COVID-19 test plus a positive chest X-ray result. COVID-19 testing to detect COVID-19 infection was performed by nasopharyngeal swab and quantitative polymerase chain reaction test. It should be noted universal COVID-19 testing for all pregnant patients at the time of hospital admission was not as the routine of the hospital, and if patients had symptoms of COVID-19 infection, had a recent trip to high-risk countries with prevalent disease, or had direct contact with someone who traveled to high-risk regions or who had COVID-19 (e.g., considered to have a high-risk exposure), they were tested when admitted to the labor and delivery unit.

Each COVID-19 case was matched to one control by gestational age. The control group of pregnant women asymptomatic or who had a negative COVID-19 test result during hospital stay were randomly selected from the medical records by an investigator (SH), who was not involved in statistical analysis. Matching was based age of the mother and gestational age of cases and termination of pregnancy. Blood test results were also retrieved from medical records. All patient data were extracted from the medical history and presented in detail in the measurement section.

### Our hospital policy during the COVID-19 pandemic

Based on our hospital policy, it should be noted, due to our limitation of conduction (negative pressure isolation delivery room), our pregnant women with confirmed COVID-19 permitted to vaginal delivery in a single isolated delivery room and strict protection measures without birthing partners present. Moreover, to reduce the possible risk of neonatal infection caused by mother-to-child contact, neonates delivered by pregnant women with confirmed or suspected COVID-19 have their umbilical cords cut and cleaned as early as possible to reduce exposure time. In the present study, the neonates delivered by pregnant women with a clinical diagnosis of COVID-19 were isolated from their mothers and admitted to the NICU immediately after birth for further observation. If routine laboratory tests were normal, throat swab tests for the nucleic acid of COVID-19 were negative twice (at least 24 h apart), and no signs of pneumonia were detected on chest radiograph, they could be discharged from the NICU. Breastfeeding was not permitted at the beginning because the infant7s and their mothers were isolated separately. Artificial feeding was provided for the infants isolated in the NICU for the first few days. The mothers were asked to maintain milk secretion by using breast pumps.

### Measures

1. COVID-19 symptoms including laboratory evaluations and clinical symptoms: Maternal symptoms include fever (defined as the temperature of > 100.4 °F), cough, shortness of breath, chest pain, diarrhea, myalgias, and sore throat. Laboratory evaluation included the results of routine complete blood counts and comprehensive metabolic panels (the latter when ordered for clinical purposes). Laboratory findings including leukocytosis (white blood cell count > 11 × 109/l), lymphopenia (lymphocyte count < 1.0 × 109/l), thrombocytopenia, and increased C-reactive protein (CRP) (10 mg/l) was considered COVID-19 laboratory findings. Clinical management had supplemental oxygen, hydroxychloroquine, remdesivir, antibiotic agents, bronchodilators, mechanical ventilation, steroids, and ICU admission.
2. Maternal demographics: Age, gravidity, parity, body mass index before pregnancy, and medical comorbidities
3. Maternal outcomes: Preeclampsia, venous thromboembolism, antepartum admission (defined as hospital admission for obstetrical or non-obstetrical indications for inpatient management for > 48 h), ICU admission, need for mechanical ventilation, supplemental oxygen, maternal death, preterm delivery (defined as delivery before 37-week gestation), mode of delivery (cesarean or vaginal delivery), intrauterine fetal dead, length of hospital stay, and chorioamnionitis
4. Neonatal outcomes: Respiratory distress syndrome (RDS; defined as the need for supplemental oxygen and the presence of typical radiographic findings in the absence of other causes for respiratory distress), 5-min Apgar score of < 7, neonatal death, birthweight, neonatal ICU (NICU) admission, and length of hospital stay

### Data analysis

We used descriptive and analytic statistics using SPSS 21(SPSS, Chicago, IL, USA) in the present study. Data are presented as mean (standard deviation) for the quantitative and *n* (%) for the qualitative variable. To compare groups, the *T* test, Mann-Whitney test, *χ*^2^, and Fisher test were used. The normality of the distributions was tested using the Kolmogorov-Smirnov test. Logistic regression was used to determine the associations between exposure to COVID-19 and adverse maternal and neonatal outcomes. Analyses were adjusted for confounders, including advanced maternal age, obesity, and comorbid medical problems. A significance level of 0.05 was acceptable. There were no missing values. Therefore, no missing imputation technique was used. The preparation of this manuscript was in accord with the STROBE guidelines for observational studies.

## Results

### The study samples

In total, 110 pregnant women participated in the study. Socio-demographic and clinical characterizes of patients are presented in Table 1. There were no significant differences between the two groups regarding the characteristics mentioned above (*p* > 0.05). All of the patients in the two groups had singleton pregnancies. In the present study, 10, 7, and 38 patients in each group were in the first, second, and third trimesters of pregnancy, respectively. However, the comorbidities during and before pregnancy were more prevalent in COVID-19 than the control group, but the difference was not statistically significant (Table 2).

**Table 1:** Socio-demographic and clinical characteristic of the patients

Variable		Case, *n* = 55	Control, *n* = 55	*p* value^a^
Age (year)^a^		30.45 ± 4.22	31.02 ± 3.63	0.49
BMI^a^		26.05 ± 3.42	24.3 ± 2.39	0.46
Occupation^b^	Occupied	5 (9.09)	3 (5.45)	0.71
	Housewife	50 (90.90)	52 (94.54)	
Nulliparous^b^		15 (27.27)	12 (21.81)	0.50
Smoking status^b^		1 (1.81)	2 (3.63)	0.55

^a^Mean (SD), *T* test

^b^*N* (%), *χ*^2^ or Fisher test

**Table 2:** Comorbidities during and before of pregnancy

Variable^a^		Case, *n* = 55	Control, *n* = 55	*p* value^a^
Pre-pregnancy	Hypertension	3 (5.45)	2 (3.63)	0.64
	Diabetes	5 (9.09)	2 (3.63)	0.24
	Asthma	3 (5.45)	0	0.09
	Previous C/S	9 (16.36)	11 (20)	0.46
During pregnancy	Gestational diabetes	4 (7.27)	2 (3.63)	0.40
	Gestational hypertension	2 (3.63)	1 (1.81)	0.55

^a^*N* (%), *χ*^2^ or Fisher test

### Clinical features, laboratory findings, and management of COVID-19

The most prevalent symptoms related to COVID-19 were fever (69.09%) and cough (58.18%). Less common symptoms included fatigue, diarrhea, shortness of breath, sore throat, and myalgia (Table 3). Based on the findings of the present study, the laboratory profile of pregnant women with COVID-19 is similar to that of the control group except for C-reactive protein concentration, ALT, and AST (*p* < 0.05) (Table 4).

**Table 3:** Clinical characteristics of case group

Variable		Case (*n* = 55), *N* (%)
**Clinical manifestations**	Fever	38 (69.09)
	Cough	32 (58.18)
	Shortness of breath	22 (40)
	Diarrhea	5 (9.09)
	Sore throat	10 (18.18)
	Headache	15 (27.27)
	Fatigue	18 (32.72)
	Myalgia	20 (36.36)
**Management of COVID-19 pneumonia**	Hydroxychloroquine/chloroquine	32 (58.18)
	Antivirus therapy	20 (36.36)
	Antibiotic therapy	25 (45.45)
	High flow oxygen	5 (9.09)
	Admitted to ICU	2 (3.63)

**Table 4:** Laboratory characteristics

Variable^a^	Case, *n* = 55	Control, *n* = 55	*p* value^a^
White blood cell count (× 10^9^cells/L)	8.92 ± 1.29	9.05 ± 0.75	0.48
Lymphocyte count (× 10^9^ cells/L)	1.12 ± 0.39	1.82 ± 0.7	0.26
C-reactive protein concentration (mg/L)	23.4 ± 1.89	6.82 ± 0.71	0. 01
ALT (U/L)	27.1 ± 1.26	18.42 ± 0.98	0.05
AST (U/L)	28.4 ± 0.75	19.06 ± 1.10	0.03
Hemoglobin (g/dL)	11.4 ± 2.02	11.2 ± 1.56	0.48
Hematocrit (%)	34.2 ± 4.01	34.51 ± 3.41	0.49
Blood urea nitrogen (mmol/L)	2.74 ± 0.51	2.84 ± 1.02	0.42
Creatinine (μmol/L)	47.31 ± 1.04	49.44 ± 3.71	0.47

^a^Mean (SD), *T* test

Hydroxychloroquine/chloroquine (58.18%) and antibiotic therapy (45.45%) were the most prevalent management in COVID-19 patients (Table 3). One patient (1.81%) died due to maternal compromise with developed severe respiratory complication required critical care due to COVID-19 infection in the case group (data not presented).

### Maternal, obstetrics, and neonatal outcomes

There was no PPROM, IUGR, and stillbirth in any of the studied groups. Based on our findings, maternal and obstetric outcomes—neonatal in case groups—such as mode of delivery, postpartum hemorrhage, perineal resection rate, neonates’ birth weight, Apgar score, and neonatal asphyxia rate were similar to pregnant women without COVID-19. The prevalence of stillbirth was 1/20 (5%) in the control group compared to 0% in the case group (*p* = 0.62). We observed a higher incidence rate of premature delivery in COVID-19 cases (25% vs. 10%) (*p* < 0.05) (Table 5).

**Table 5:** Maternal and neonatal outcomes of the patients

Variable		Case, *n* = 55	Control, *n* = 55	*p* value^b^
Maternal	Cesarean section^b, c^	9/20 (45)	8/20 (40)	0.74
	Perineal incision^b, d^	7/11 (63.63)	5/11 (45.45)	0.39
	Abortion < 21 w^b, e^	3/14 (21.42)	2/14 (14.28)	0.62
	Amniotic pollution^b^	3/20 (15)	4/20 (20)	0.67
	Placenta abruption^b^	1/20 (5)	2/20 (10)	0.54
	IUFD^b^	0	1/20 (5)	0.62
	Postpartum hemorrhage^b^	1/20 (5)	3/20 (15)	0.29
	Preterm birth^b^	5/20 (25)	2/20 (10)	0.04
Neonate	Neonate weight ^a^	3251.04 ± 4.32	3271.59 ± 5.30	0.80
	Fetal distress^b^	2/20 (10)	1/20 (5)	0.54
	Low birth weight^b^	2/20 (10)	1/20 (5)	0.54
	Apgar in 5 min < 7^b^	3/20 (15)	4/20 (20)	0.67
	Respiratory distress^b^	3/20 (15)	1/20 (5)	0.29
	NICU admission^b^	2/20 (10)	3/20 (15)	0.63
	Neonatal dead^b^	1/20 (5)	0	0.62

^a^Mean (SD), *T* test

^b^*N* (%), *χ*^2^ or Fisher test

^c^The denominator of the deduction indicates the number of people who terminated the pregnancy

^d^The denominator of the deduction indicates the number of people who terminated the pregnancy by NV/D

^e^The denominator of the deduction indicates the number of people who were < 21 week of pregnancy

In the present study, we found that women with COVID-19 had a more than twofold increased odds of preterm labor. History of preterm delivery was also associated with high twofold odds of preterm labor (Table 6).

**Table 6:** Predictive factors of preterm labor in case group based on the result of logistic regression test

Variable	*p* value	SE	Odds ratio	95% CI	
				Lower	Upper
COVID-19	0.04	0.48	2.64	0.98	4.99
History of preterm labor	0.02	0.41	2.03	1.14	4.78

Hosmer and Lemeshow test, *p* = 0.84

## Discussion

To the best of our knowledge, this is the first comprehensive case-control study in Iran that compares maternal and neonatal outcomes in positive COVID-19 with negative pregnant women. In summary, the most prevalent symptoms related to COVID-19 were fever (69.09%) and cough (58.18%). Less common symptoms included fatigue, diarrhea, shortness of breath, sore throat, and myalgia. Based on the present study’s findings, the laboratory profile of pregnant women with COVID-19 is similar to that of the control group except for C-reactive protein concentration, ALT, and AST. Chen et al. reported the most prevalent symptoms of COVID-19 infection in nine pregnant women were fever and cough [11]. Our results replicate the findings of a previous study in pregnant women with SARS in Hong Kong, which reported that fever was the predominant symptom [2].

One of the most critical concerns of obstetricians during the outbreak of COVID-19 is whether pregnant women will be worse off. Based on our findings, maternal and obstetric outcomes—neonatal in case groups—such as mode of delivery, premature rupture of membrane, postpartum hemorrhage, perineal resection rate, birth weight of neonates, Apgar score, and neonatal asphyxia rate were similar to pregnant women without COVID-19. In a case study with ten pregnant women infected with COVID-19, five underwent emergency cesarean section due to the fetus distress (three, 30%), premature rupture of membrane (one, 10%), and stillbirth (one, 10%) even though the severity of COVID-19 in most of these patients was as mild to moderate classified [3]. Another study was performed on 16 pregnant women with COVID-19 and 45 non-infected pregnant women in the third trimester of pregnancy. The results showed no increased risk of perinatal complications in women infected with COVID-19, including severe preeclampsia, premature rupture of membranes, fetal distress, meconium-stained amniotic fluid, preterm delivery, neonatal asphyxia, and postpartum hemorrhage [4]. These conflicting results may be due to selection bias. This suggests that the effects of COVID-19 in pregnancy require further study. In the last systematic review including seventeen studies comprising 84 live births, adverse outcomes including stillbirth (1.2%), neonatal death (1.2%), preterm birth (21.3%), low birth weight (< 2500 g, 5.3%), fetal distress (10.7%), and neonatal asphyxia (1.2%) were reported. The case-control study showed no significant differences in fetal and neonatal outcomes (fetal distress [*p* = 0.668], preterm birth [*p* = 0.686], and neonatal asphyxia [*p* = 0.441]) between groups [5]. However, available data on fetal and neonatal outcomes to date only include pregnant women infected in their third trimesters, same to our study. We did not follow our mothers who were COVID-19 positive until delivery to determine whether infection in the first or second trimester would increase the risk of adverse fetal and neonatal outcomes. Therefore, future follow-up studies of COVID-19 in pregnant women in early pregnancy to evaluate vertical transmission potential are urgently needed. In our study, all the neonates we tested were negative for COVID-19, consistently with previous reports [1, 6]. However, no conclusion can be drawn from our results about the possibility of intrauterine vertical transmission.

During the COVID-19 outbreak, it is proposed that cesarean delivery under general anesthesia has been the preferred mode of delivery to ensure a controllable delivery process, avoid emergency respiratory problems, and reduce the risk of infection exposure. However, the effects of these measures have not been fully proven. In a retrospective case series of 43 pregnant women with PCR-confirmed COVID-19 infection from two American centers, most women were obese, and 18 had additional co-morbidity. Among these 43 women, 18 gave birth, ten by uncomplicated vaginal deliveries, and eight by cesareans for obstetric reasons unrelated to COVID [7]. For common type COVID-19 patients in our hospital, vaginal delivery can be chosen if the relationship between the fetal head and pelvis is good. It is estimated that vaginal delivery can be performed within a short time. Pregnant women were given nasal catheter oxygen inhalation and wore medical surgical masks in the isolation delivery room. Freedom to move may alleviate pain and promote vaginal delivery during the labor process. If necessary, episiotomy, forceps delivery, and vacuum extraction can be used. Given our study’s small sample size, the possibility of vertical transmission during vaginal delivery still cannot be ruled out. Li et al. [8] confirmed and suspected COVID-19 infection had been included as one indication for cesarean section in their hospital because there was only one negative pressure operation room suitable for airborne precautions. However, two patients had vaginal delivery in positive pressure labor rooms before being diagnosed with COVID-19 pneumonia. No transmission events occurred in the doctors and midwives, wearing a full set of personal protective equipment (N95 respirators, protective gown, coveralls, gloves, and goggles) during the delivery procedure. In the expert consensus (2020), it is recommended that delivery and delivery timing is individualized based on obstetrical indications and maternal-fetal status [10].

Our findings have shown in the case group that preterm delivery was significantly more than pregnant women without COVID-19. Yan and colleagues reported a retrospective series expanded from four previous small case series, totaling 116 pregnant women with COVID-19 pneumonia from 25 hospitals in China [1, 3, 9]. Preterm delivery before 34- and 37-week gestation occurred in 2% and 21.2% of cases: 6.9% (8/116) of the women were admitted to ICU, 5.2% (6/116) required non-invasive and 1.7% (2/116) invasive ventilation, and 0.9% (1/116) ECMO. While these results were consistent with ours, the authors concluded that “there is no evidence that pregnant women with COVID-19 are more prone to develop severe pneumonia, in comparison to nonpregnant patients.” [10]. In Knight et al. (2020) [12], over half of all women admitted with COVID-19 infection have given birth; 12% were delivered preterm solely due to maternal respiratory compromise. Almost 60% of women gave birth by cesarean section; most cesarean deliveries were for indications other than the maternal center due to COVID-19 infection. However, during the COVID-19 study (2021), Danish premature birth rates concluded COVID-19 lockdown has drastically changed their lives by changing our working environment, reducing physical interactions, and increasing our focus on hygiene. This unusual situation is likely to have influenced several risk factors for premature birth [13]. Prematurity is a complex and challenging pathophysiological condition associated with an increased risk of long-term morbidity and mortality. It is the leading cause of death in children under 5 years of age [13]. Prevalence of preterm birth is different among regions and countries. Worldwide, an estimated 11.1% of all live births were born preterm in 2010 [14]. Based on the World Health Organization’s 2011 report, the prevalence of preterm delivery in 184 countries was between 5 and 18%, of which 60% of these deliveries occurred in Africa and South Asia. In low-income countries, on average, 12% of babies are born prematurely; in contrast, this rate is 9% in high-income countries [15]. However, in some high-income countries (such as the USA with 12% and Australia with 10.9%), premature birth is considerable [16]. The overall estimated prevalence of preterm in Iran was 9.2%. Based on some studies in Iran, low general health status, history of disease during pregnancy, family history of prematurity, previous preterm labor, history of last neonatal death, periodontal disease, decreased amniotic fluid, multiple pregnancies, infertility, and cervical incompetence were identified as risk factors of preterm labor [17]. Our regression analysis found that women with COVID-19 had a more than twofold increased odds of preterm labor. History of preterm delivery was also associated with high twofold odds of preterm labor.

Our study has several strengths, including greater sample size and the inclusion in a control group design for comparison. There are some limitations. First, most of the included patients presented as mild to moderate, which limited the interpretation of results. Second, due to the retrospective study’s nature, we could not test samples of the placenta, amniotic fluid, cord blood, and vaginal mucosa, which weakened our conclusion of no vertical transmission potential. Third, we did not take throat swab samples of all the newborns to check for COVID-19 infection. But more case-control studies are needed to ascertain the results. Serological studies and those using prospective data to identify women with either confirmed or presumed mild infection in pregnancy will be essential to fully assess potential impacts such as congenital anomalies, miscarriage, or intrauterine fetal growth restriction.

## Conclusions

We observed a higher incidence rate of premature delivery in COVID-19 cases. Women with COVID-19 had a more than twofold increased odds of preterm labor. Considering prematurity has high morbidity and is regarded as the primary cause of mortality in children under 5 years old, more case-control studies are needed to ascertain the results.

## Data Availability

The primary data for this study is available from the authors (FB) on direct request.
